# Supplementary material for: Immobility responses are induced by photoactivation of single glomerular species responsive to fox odour TMT
Source: Nat Commun. 2017 Jul 7;8:16011. doi: 10.1038/ncomms16011 (PMC5504302; doi:10.1038/ncomms16011)
Supplement: Supplementary Information [file ncomms16011-s1.pdf]

Type of file: PDF
Size of file: 0 KB
Title of file for HTML: Supplementary Information
Description: Supplementary Figures and Supplementary Table

Type of file: MOV
Size of file: 0 KB
Title of file for HTML: Supplementary Movie 1
Description: Three-dimensional computer graphics for Egr1 expression in the AON. KI mice of Olfr1019-ChRWR were photo-illuminated (Photo) with a 2 Hz light for 30 min. Control mice (WT) were exposed to 10% TMT for 30 min. Brain sections were analyzed by immunohistochemistry for the activity dependent expression of Egr1, an immediate-early gene product. Pictures of fluorescence-labeled Egr1 expression in the 20 µm sections were taken every 100µm. The volume rendering software (VCAT5) was used to incorporate the pictures and to process the images. Movie is shown for the AON.

Type of file: MOV
Size of file: 0 KB
Title of file for HTML: Supplementary Movie 2
Description: Three-dimensional computer graphics for Egr1 expression in the APC and OT. KI mice of Olfr1019-ChRWR were photo-illuminated (Photo) with a 2 Hz light for 30 min. Control mice (WT) were exposed to 10% TMT for 30 min. Brain sections were analyzed by immunohistochemistry for the activity dependent expression of Egr1, an immediate-early gene product. Pictures of fluorescence-labeled Egr1 expression in the 20 µm sections were taken every 100µm. The volume rendering software (VCAT5) was used to incorporate the pictures and to process the images. Movie is shown for the APC and OT.

Type of file: MOV
Size of file: 0 KB
Title of file for HTML: Supplementary Movie 3
Description: Photo-induced responses of the KI mice (5 sec illumination). Photo-illumination, 2 Hz, 250 msec pulses, was given for 5 sec to KI and WT mice. Behaviors after illumination are compared between the KI (Olfr1019-ChRWR) and WT mice.

Type of file: MOV
Size of file: 0 KB
Title of file for HTML: Supplementary Movie 4
Description: Photo-induced responses of the KI mice (1 sec illumination). Photo-illumination, 2 Hz, 250 msec pulses, was given for 1 sec to KI and WT mice. Behaviors after illumination are compared between the KI (Olfr1019-ChRWR) and WT mice.

Type of file: PDF
Size of file: 0 KB
Title of file for HTML: Peer Review File
Description:

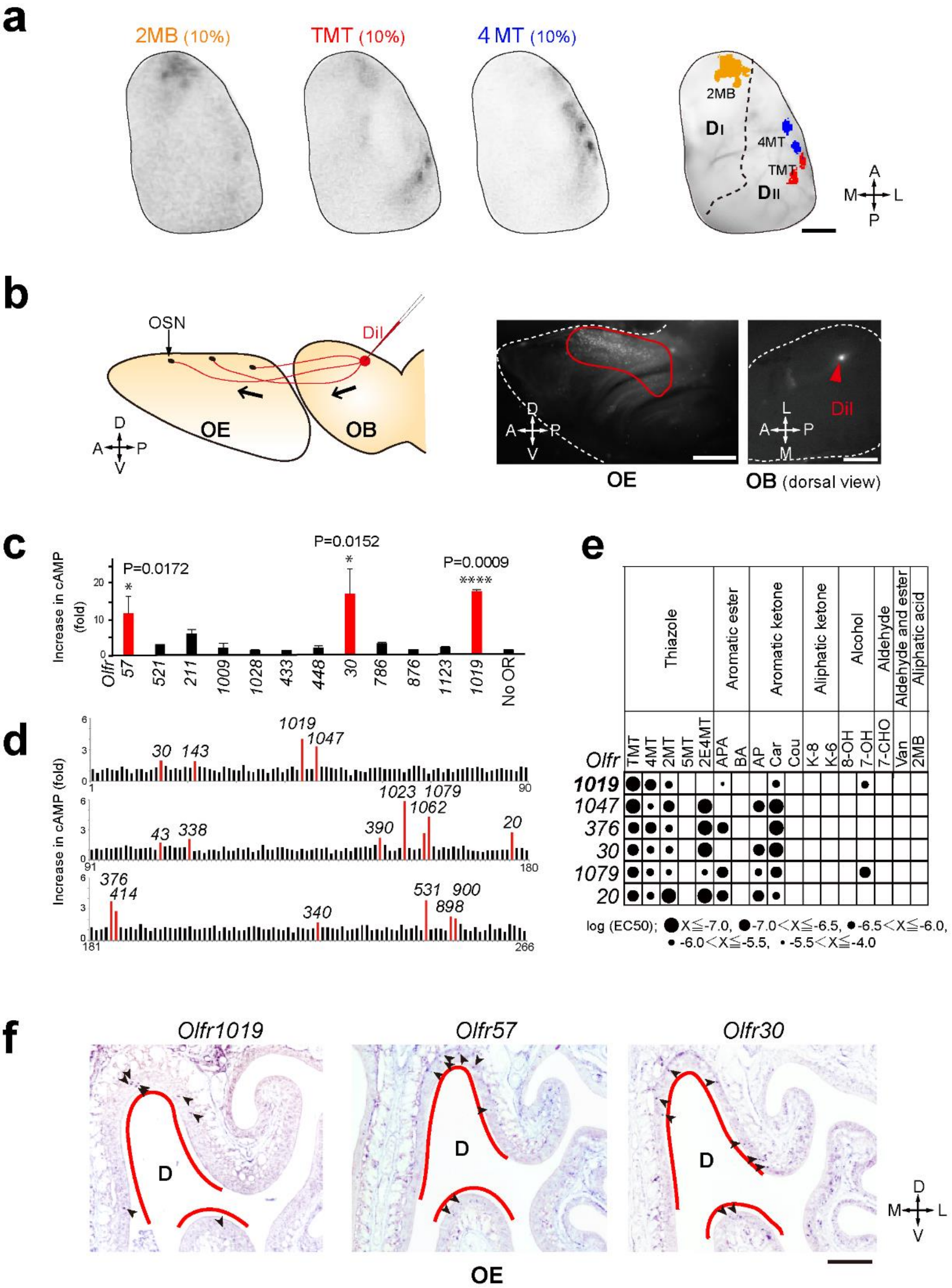
a
2MB (10%)
TMT (10%)
4 MT (10%)
2MB
DI
4MT
TMT
DII
A
M
L
P
b
OSN
DiI
OE
OB
D
A
P
V
L
M
OE
OB (dorsal view)
c
Increase in cAMP (fold)
P=0.0172
P=0.0152
P=0.0009
*
*
****
Olfr
57
521
211
1009
1028
433
448
30
786
876
1123
1019
No OR
d
Increase in cAMP (fold)
30
143
1019
1047
43
338
390
1023
1079
1062
20
376
414
340
531
900
898
1
90
91
180
181
266
e
Thiazole
Aromatic ester
Aromatic ketone
Aliphatic ketone
Alcohol
Aldehyde
Aldehyde and ester
Aliphatic acid
Olfr
TMT
4MT
2MT
5MT
2E4MT
APA
BA
AP
Car
Cou
K-8
K-6
8-OH
7-OH
7-CHO
Van
2MB
1019
1047
376
30
1079
20
log (EC50); X≦-7.0, -7.0＜X≦-6.5, -6.5＜X≦-6.0,
-6.0＜X≦-5.5, -5.5＜X≦-4.0
f
Olfr1019
Olfr57
Olfr30
D
OE
M
L
V

**Supplementary Figure. 1 Isolation of TMT-responsive *OR* genes.**

(**a**) Optical imaging of the dorsal-OB. Activation patterns are compared for three different odorants, 2MB (10%), TMT (10%), and 4MT (10%). Schematic diagrams of imaging patterns are shown (right). A, anterior; P, posterior; M, medial; and L, lateral. Scale bar is 500 μm. (**b**) DiI staining of the OE and OB. DiI was injected into the glomeruli responded to 10% TMT to stain the connecting OSN axons and cell bodies. Scale bars are 500 μm. OB, olfactory bulb; OE, olfactory epithelium; A, anterior; P, posterior; M, medial; and L, lateral. (**c**) The cAMP-induced luciferase assay. Twelve *OR* genes repeatedly isolated from the DiI-labeled OSNs were analyze for their interactions to TMT by the cAMP dependent luciferase assay. Three ORs (*Olfr57*, *30*, and *1019*) were significantly activated by 1mM TMT and subjected to further analyses. Asterisks indicate T-test. Error bars are ±SEM. **p* <0.05, ** *p* <0.01, *** *p* <0.001. (**d**) High-throughput screening of TMT-responsive ORs. Separate from the DiI-cloning, a total of 266 published ORs (Supplementary Table 1b) were examined for their binding to TMT. Seventeen ORs (shown in red) demonstrated the significant binding to 1 mM TMT by the luciferase assay. All values in mean ±SEM. (**e**) Ligand selectivity of candidate ORs. Six D-zone ORs that demonstrated high affinity to TMT were analyzed for their binding to 17 different odorants; TMT, 2, 4, 5-trimethyl thiazoline; 4MT, 4-methyl-thiazoline; 2MT, 2methyl-thiazoline; 5MT, 5-methyl-thiazoline; 2E4MT, 2-ethyl-4 methyl-thiazoline; APA, allyl-phenyl-acetate; BA, butyl-acetate; AP, acetophenone; Car, carvone; Cou, coumarine; K-8, octanone; K-6, hexanone; 8-OH, octanol; 7-OH, heptanol; 7-CHO, heptanaldehyade; Van, vanillin; 2MB, 2-methyl-butyric acid. Binding affinities are shown in are shown in various sizes of dots. (**f**) *In situ* hybridization of the OE. D zone-specific *OR* genes, *Olfr1019*, *57*, and *30*, were analyzed. Red lines indicate expression areas in the dorsal zone. Scale bar is 200 μm. Arrowheads indicate hybridization signals. D, dorsal; V, ventral; M, medial; and L, lateral.

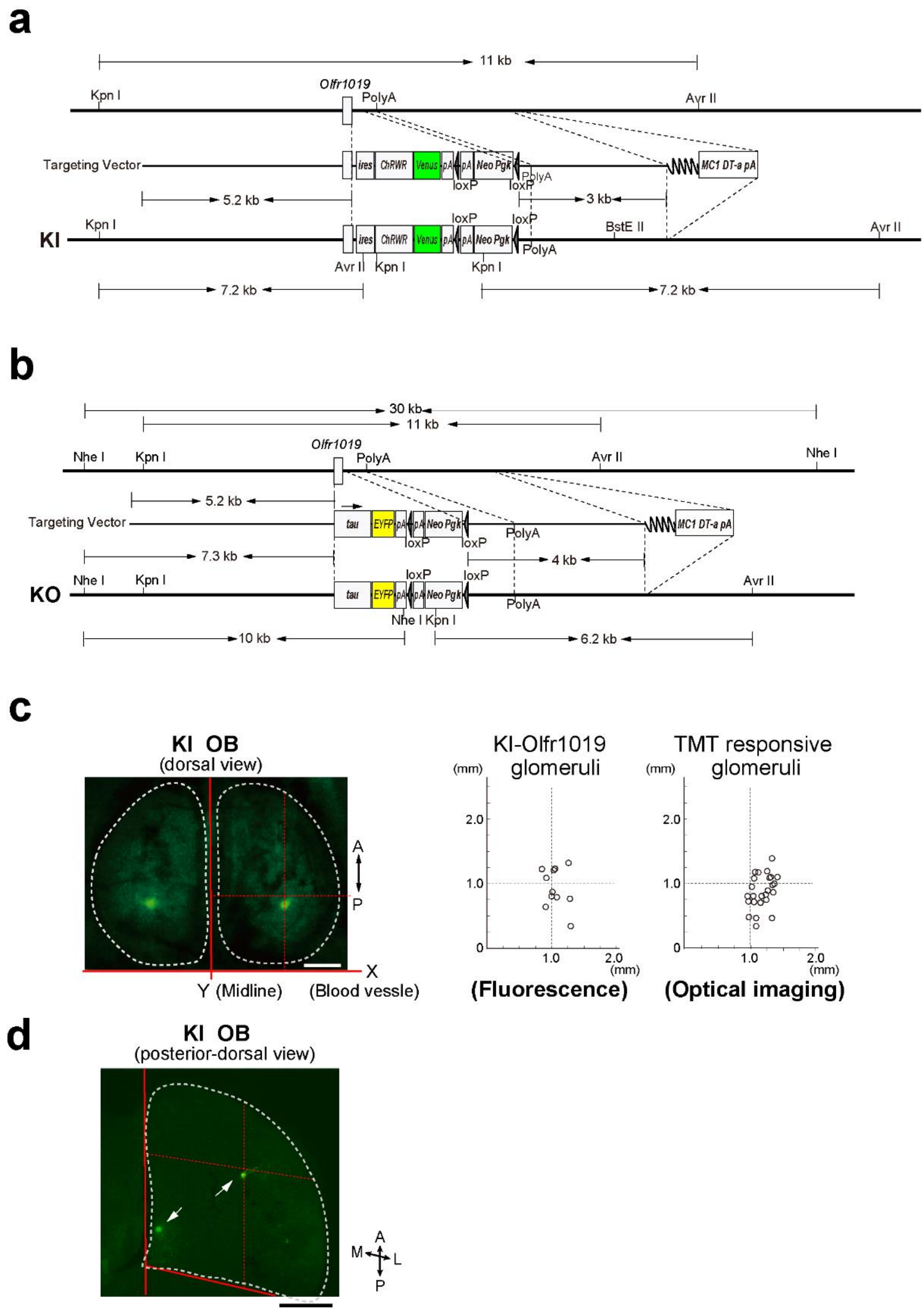
a
11 kb
Kpn I
Olfr1019
PolyA
Avr II
Targeting Vector
ires
ChRWR
Venus
pA
Neo Pgk
loxP
MC1 DT-a pA
5.2 kb
3 kb
KI
BstE II
Avr II
Kpn I
7.2 kb
b
30 kb
11 kb
Nhe I
Kpn I
Olfr1019
PolyA
Avr II
Nhe I
5.2 kb
Targeting Vector
tau
EYFP
pA
Neo Pgk
loxP
MC1 DT-a pA
7.3 kb
4 kb
KO
10 kb
6.2 kb
c
KI OB
(dorsal view)
A
P
X
Y (Midline)
(Blood vessle)
KI-Olfr1019 glomeruli
TMT responsive glomeruli
(mm)
2.0
1.0
0
(Fluorescence)
(Optical imaging)
d
KI OB
(posterior-dorsal view)
A
M
L
P

**Supplementary Figure. 2 KI and KO mice for the *OR1019* locus.**

(**a**) and (**b**) Plasmid constructs for the KI and KO mice. In the KI-allele of *Olfr1019*, coding sequences for channel-rhodopsin wide receiver (ChRWR) and a fluorescent marker (Venus) are inserted with the *ires* sequence. In the KO allele, the coding sequence of *Olfr1019* was replaced with that of *tau-EYFP*. Restriction-enzyme cleavage sites are shown for *Kpn*I, *Bst*EII, *Avr*II, and *Nhe*I. PolyA sites are also shown. (**c**) Locations of Olfr1019-KI glomeruli (green) were determined based on the midline (Y axis) and blood vessels in the boundary of the OB and neocortex (X axis). n=11. TMT-responsive glomeruli were identified by optical imaging. n=25. Scale bar is 500 μm. (**d**) Detection of Olfr1019 glomeruli in the KI mouse. Olfr1019 gives rise to two glomeruli, one is in the medial and the other is in the dorsal OB. Both are localized in the posterior region of the OB.

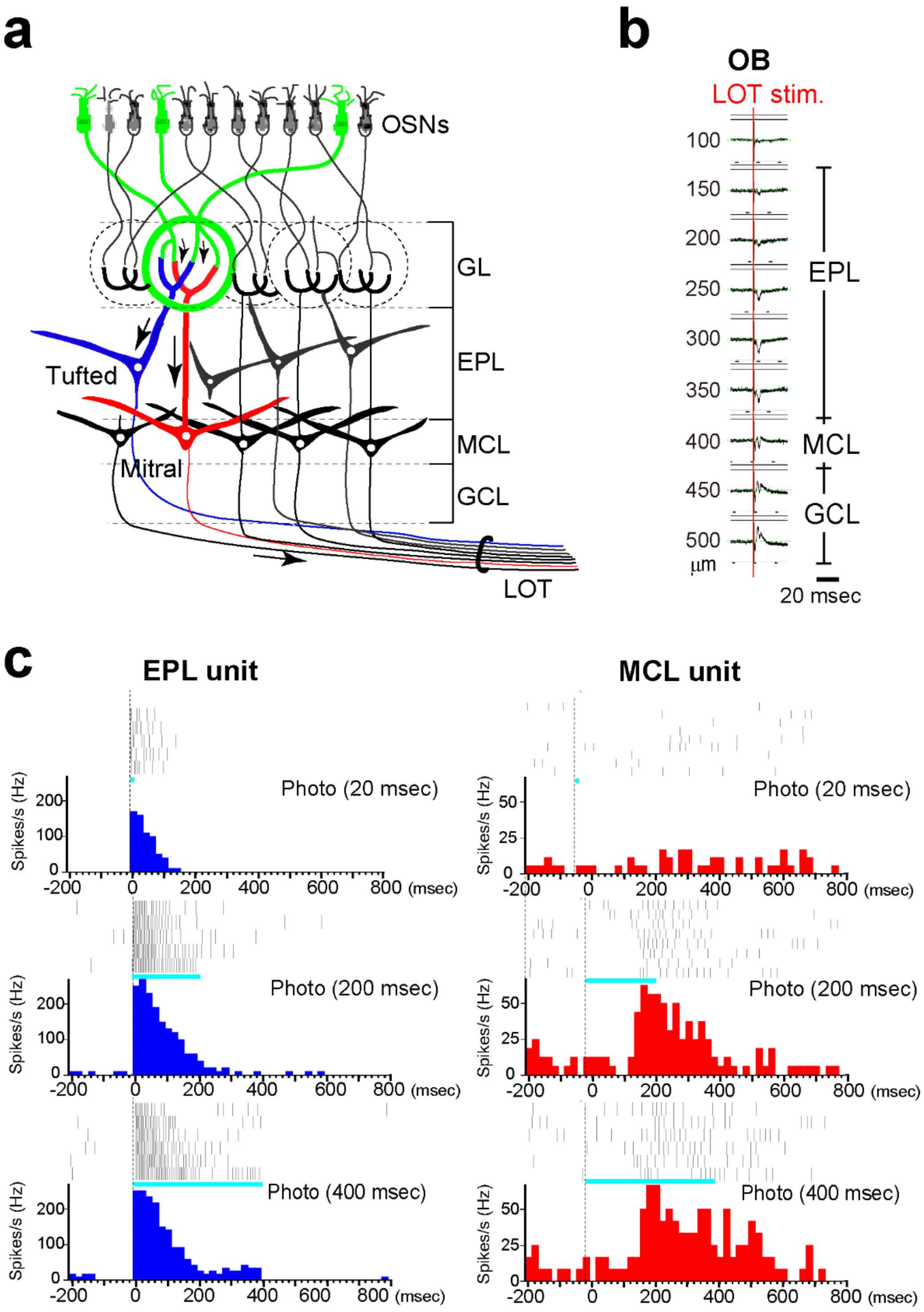
a
OSNs
GL
EPL
MCL
GCL
Tufted
Mitral
LOT
b
OB
LOT stim.
100
150
200
250
300
350
400
450
500
µm
EPL
MCL
GCL
20 msec
c
EPL unit
MCL unit
Photo (20 msec)
Photo (200 msec)
Photo (400 msec)
Spikes/s (Hz)
(msec)

**Supplementary Figure. 3 Single-unit recordings in the KI-OB**.

(**a**) A schematic diagram of layer structure in the OB. (**b**) A local field potential (LFP) in the OB. LFP was recorded in the different layers. Location of a microelectrode tip was determined by the configuration of field potentials of mitral and tufted cells. Action potentials were evoked by electric stimulation of the lateral olfactory tract (LOT). EPL, external plexiform layer; MCL, mitral cell layer; GCL, granule cell layer. (**c**) Light-evoked spike discharges in the EPL and MCL units detected by the single unit recordings. Spike timing and frequencies are shown in raster plots of spike discharges and peristimulus-time histograms. Durations of photo-illumination are indicated by cyan bars. n=5-8.

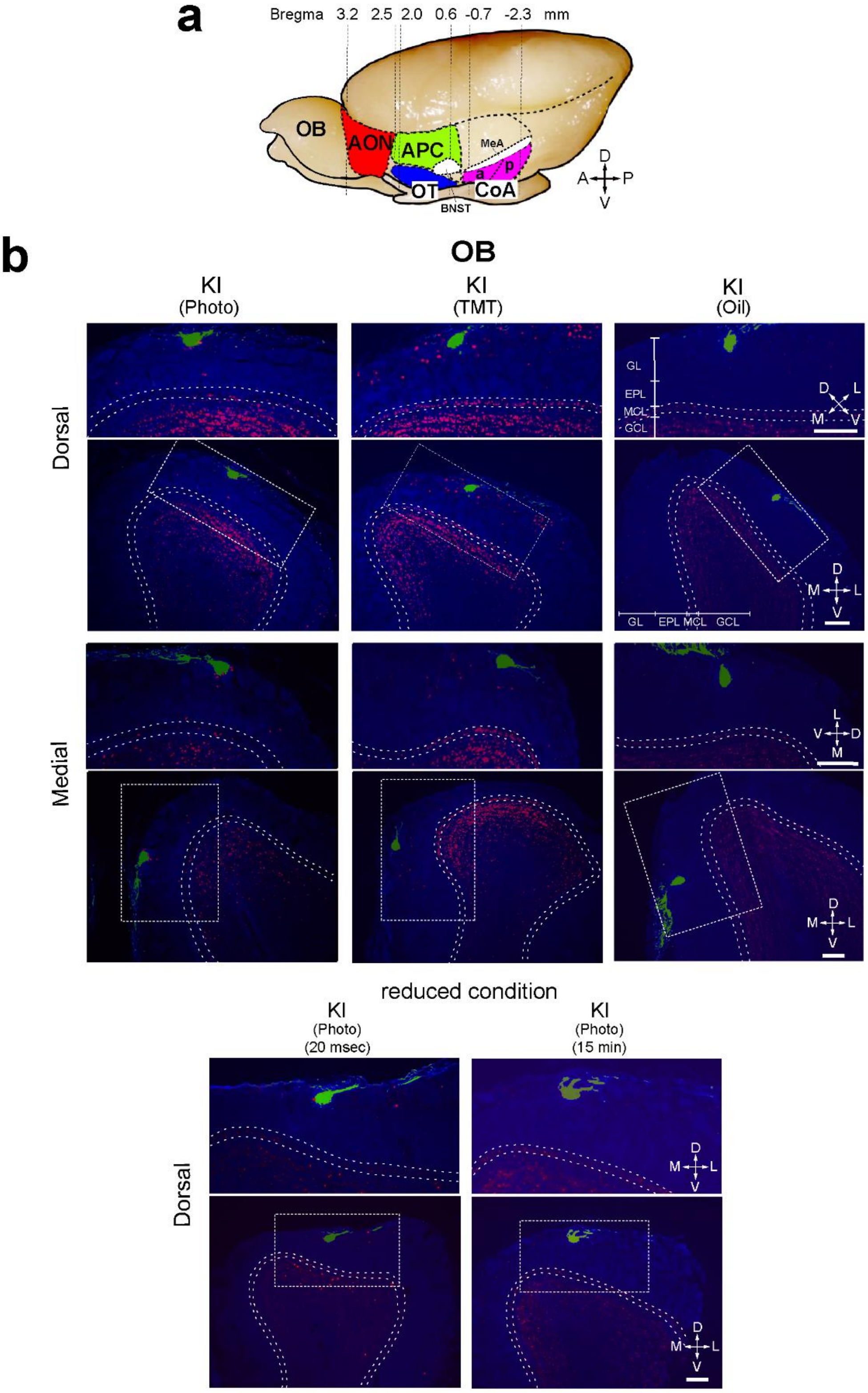
a
Bregma 3.2 2.5 2.0 0.6 -0.7 -2.3 mm
OB
AON
APC
MeA
a
p
OT
CoA
BNST
D
A
P
V
b
OB
KI (Photo)
KI (TMT)
KI (Oil)
Dorsal
GL
EPL
MCL
GCL
D L M V
D M L V
GL EPL MCL GCL
Medial
L V D M
D M L V
reduced condition
KI (Photo) (20 msec)
KI (Photo) (15 min)
Dorsal
D M L V
D M L V

**C**

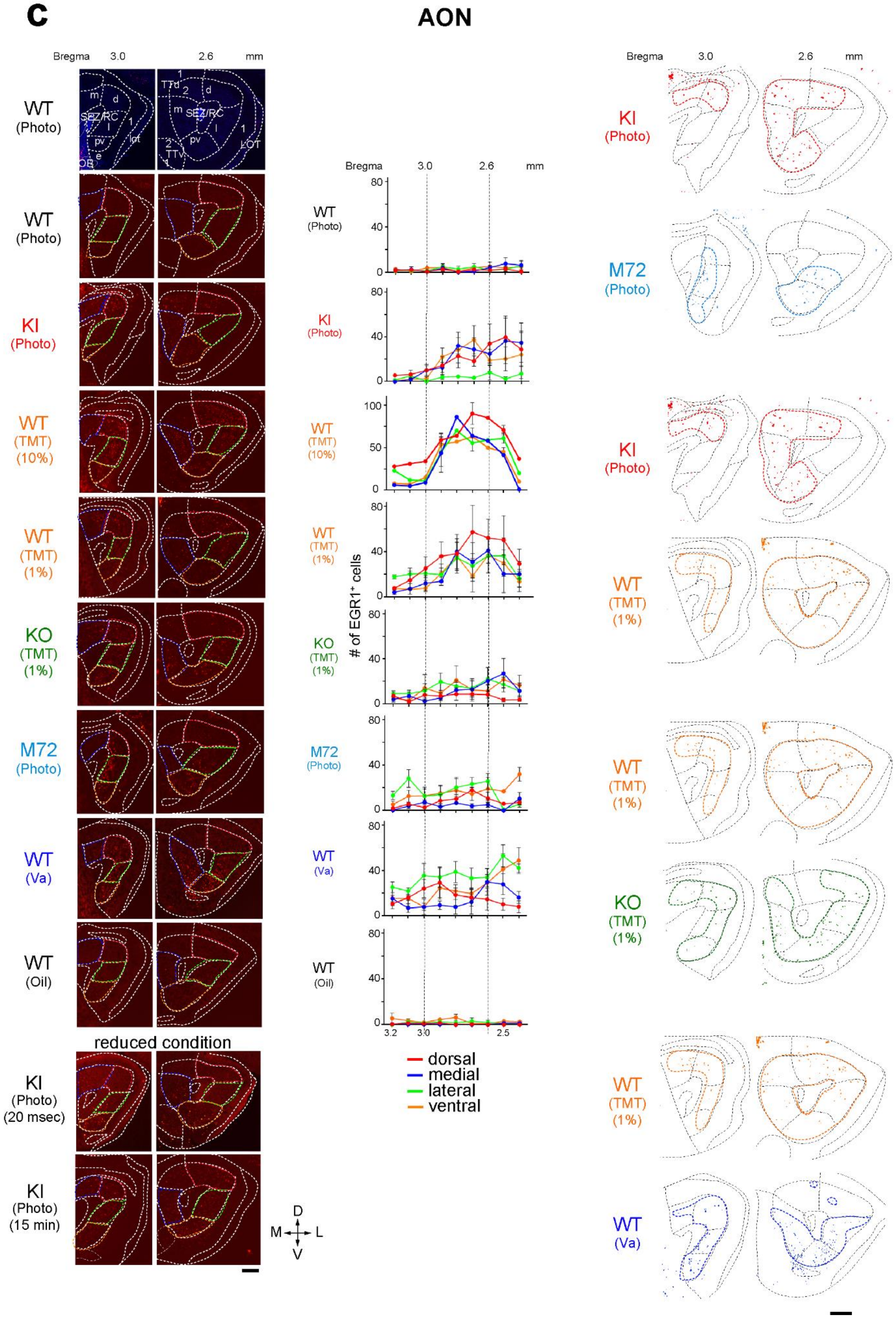
AON
Bregma 3.0 2.6 mm
WT (Photo)
KI (Photo)
WT (TMT) (10%)
WT (TMT) (1%)
KO (TMT) (1%)
M72 (Photo)
WT (Va)
WT (Oil)
reduced condition
KI (Photo) (20 msec)
KI (Photo) (15 min)
# of EGR1+ cells
dorsal
medial
lateral
ventral
D M L V

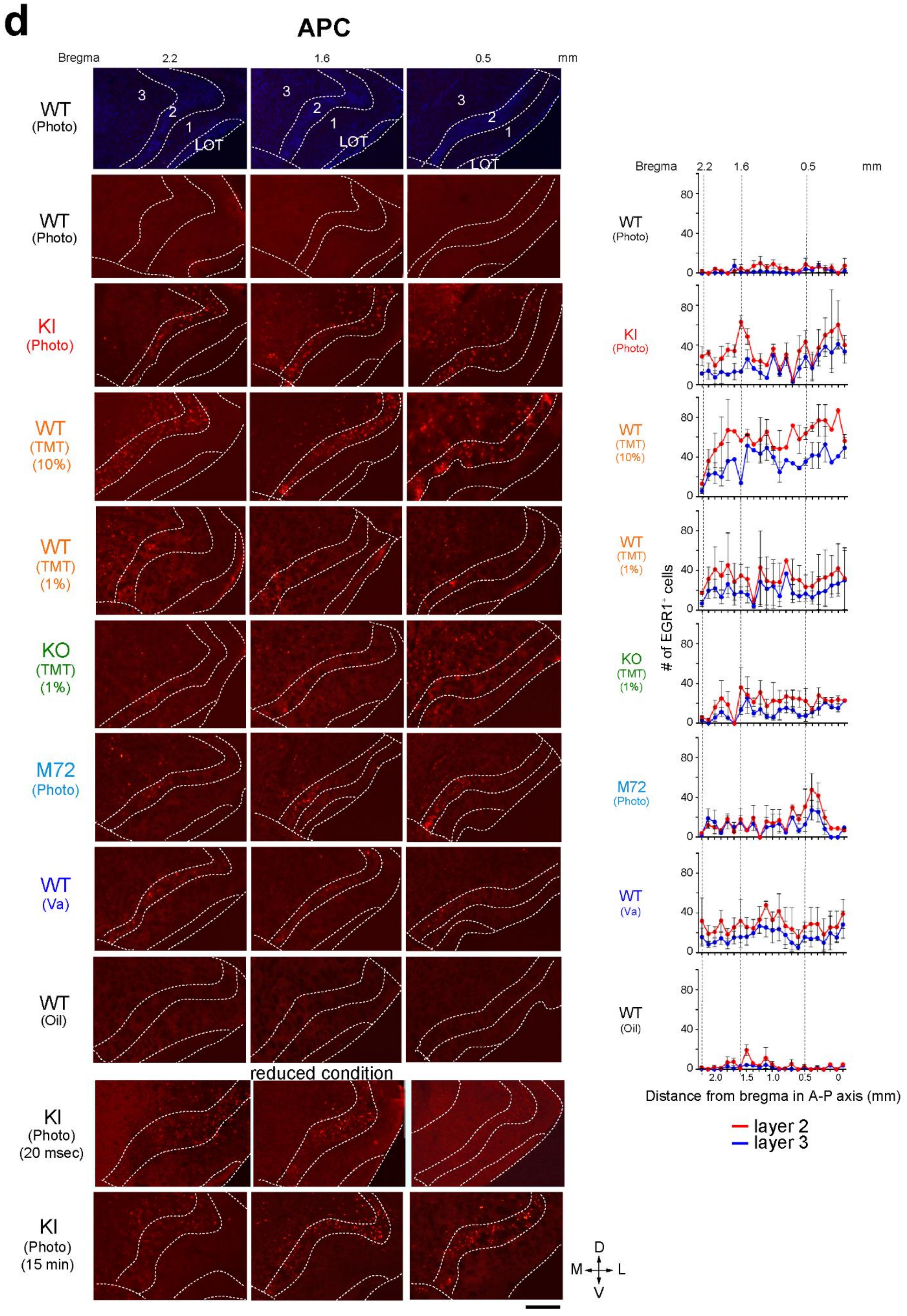

d
APC
Bregma
2.2
1.6
0.5
mm
3
2
1
LOT
WT
(Photo)
KI
(Photo)
WT
(TMT)
(10%)
WT
(TMT)
(1%)
KO
(TMT)
(1%)
M72
(Photo)
WT
(Va)
WT
(Oil)
reduced condition
KI
(Photo)
(20 msec)
KI
(Photo)
(15 min)
D
M
L
V
# of EGR1⁺ cells
80
40
0
2.0
1.5
1.0
0.5
0
Distance from bregma in A-P axis (mm)
layer 2
layer 3

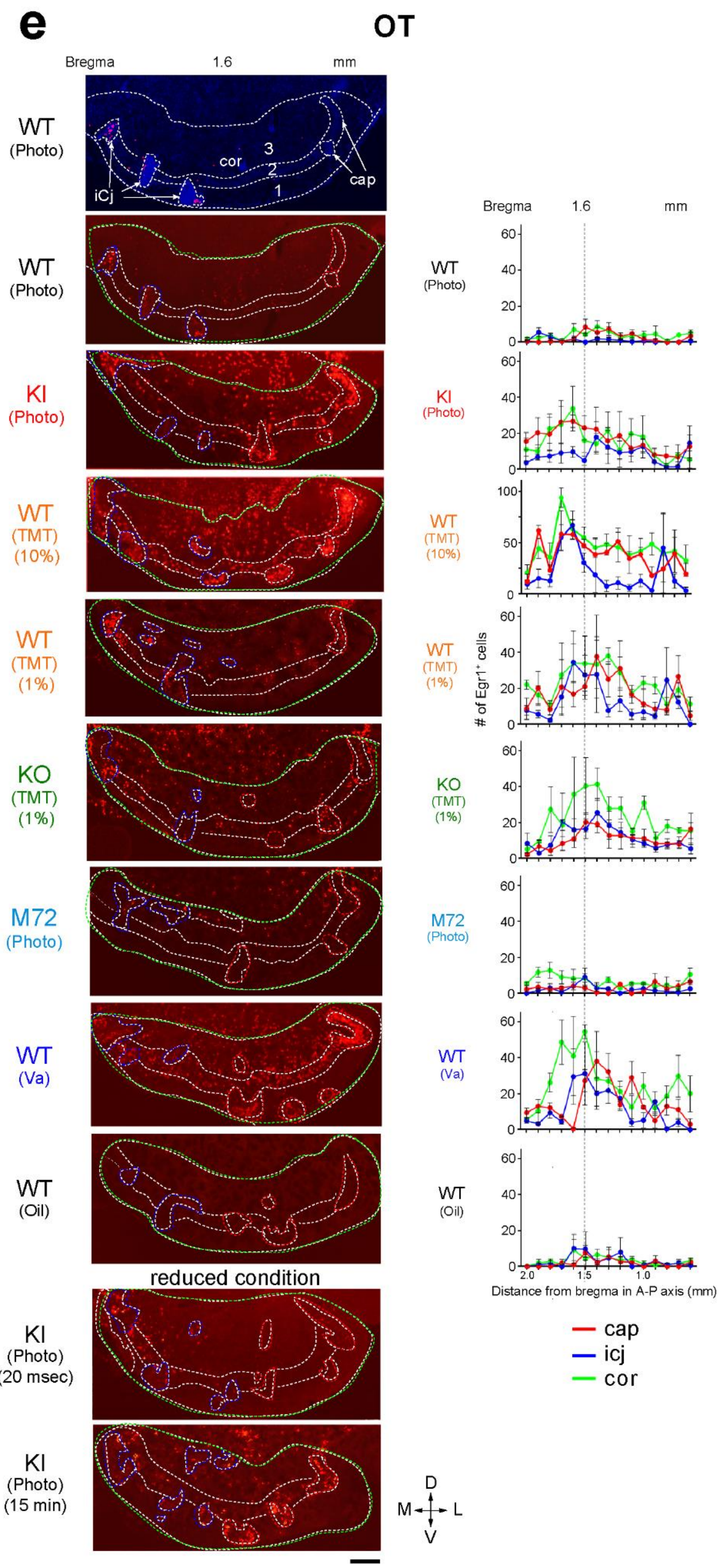

e
OT
Bregma
1.6
mm
WT
(Photo)
cor
3
2
1
cap
iCj
WT
(Photo)
KI
(Photo)
WT
(TMT)
(10%)
WT
(TMT)
(1%)
KO
(TMT)
(1%)
M72
(Photo)
WT
(Va)
WT
(Oil)
reduced condition
KI
(Photo)
(20 msec)
KI
(Photo)
(15 min)
D
M
L
V
# of Egr1+ cells
Distance from bregma in A-P axis (mm)
cap
icj
cor

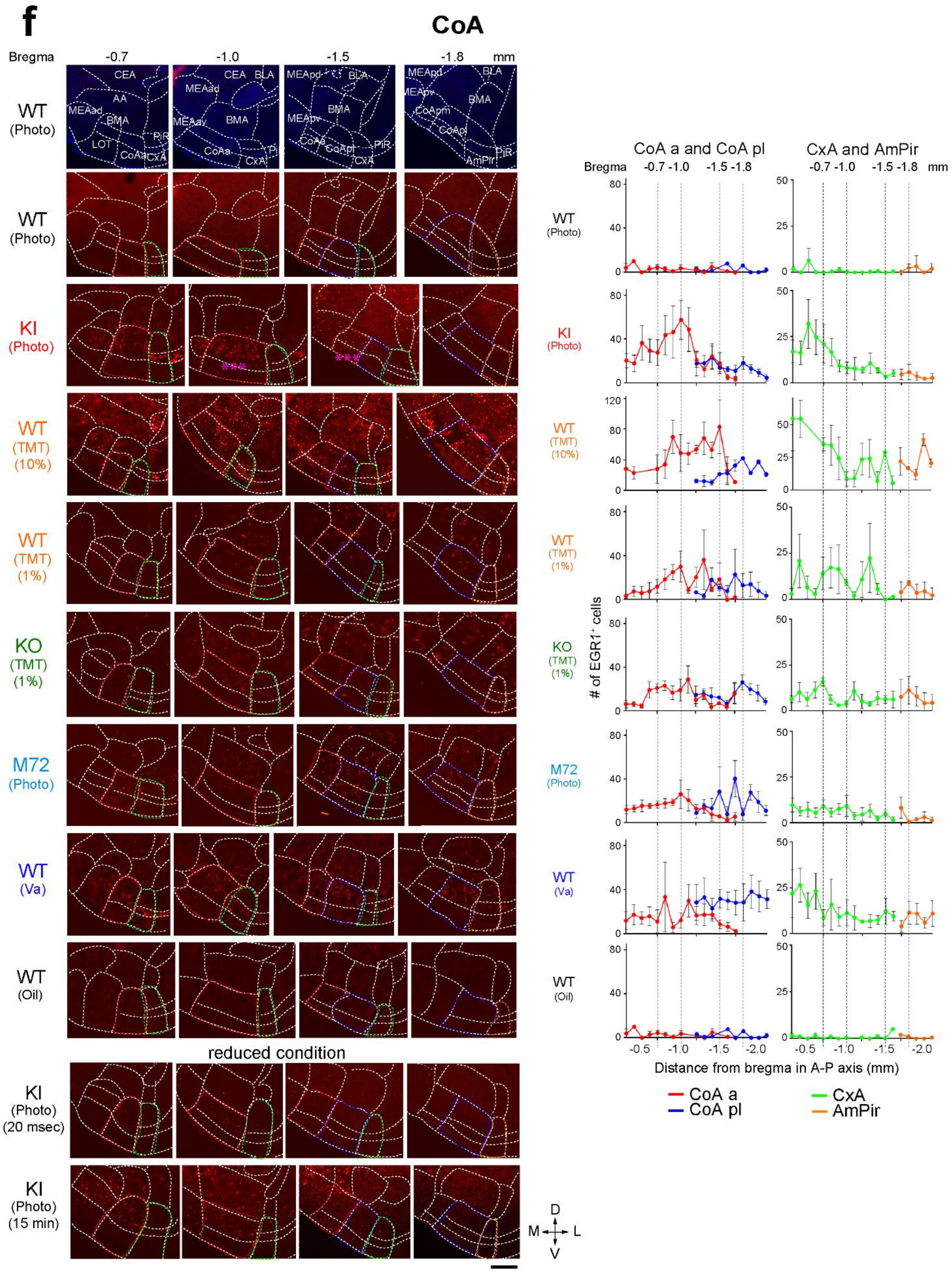
f
CoA
Bregma
-0.7
-1.0
-1.5
-1.8
mm
WT (Photo)
CEA
AA
MEAad
BMA
LOT
CoAa
CxA
PIR
BLA
MEAav
Pir
MEApd
MEApv
CoApl
CoApm
AmPir
WT (Photo)
KI (Photo)
WT (TMT) (10%)
WT (TMT) (1%)
KO (TMT) (1%)
M72 (Photo)
WT (Va)
WT (Oil)
reduced condition
KI (Photo) (20 msec)
KI (Photo) (15 min)
D
M
L
V
CoA a and CoA pl
CxA and AmPir
Bregma -0.7 -1.0 -1.5 -1.8 mm
# of EGR1⁺ cells
Distance from bregma in A-P axis (mm)
CoA a
CoA pl
CxA
AmPir

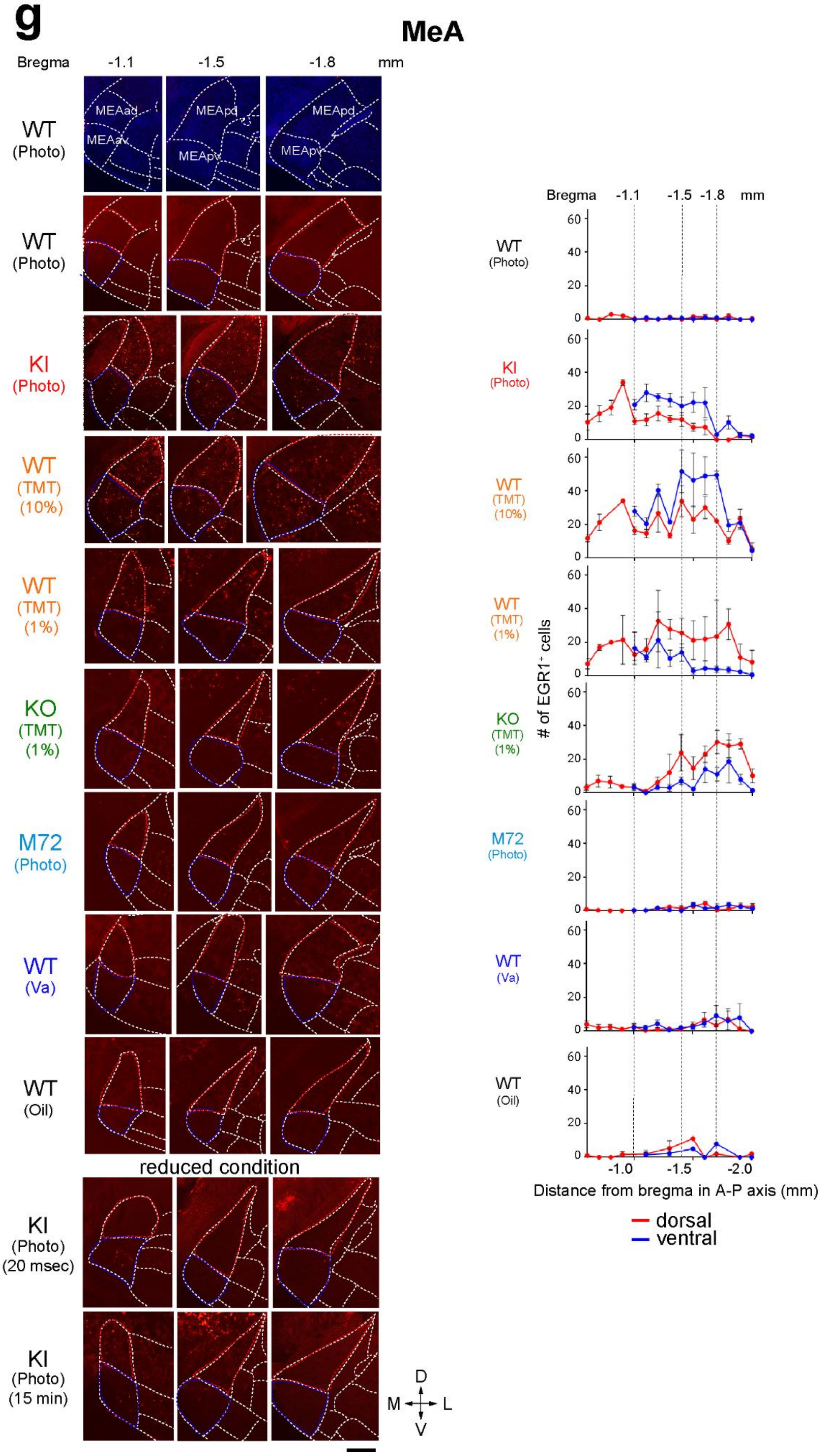
g
MeA
Bregma -1.1 -1.5 -1.8 mm
MEAad
MEAav
MEApd
MEApv
WT (Photo)
WT (Photo)
KI (Photo)
WT (TMT) (10%)
WT (TMT) (1%)
KO (TMT) (1%)
M72 (Photo)
WT (Va)
WT (Oil)
reduced condition
KI (Photo) (20 msec)
KI (Photo) (15 min)
D
M
L
V
Bregma -1.1 -1.5 -1.8 mm
# of EGR1+ cells
Distance from bregma in A-P axis (mm)
-1.0 -1.5 -2.0
dorsal
ventral

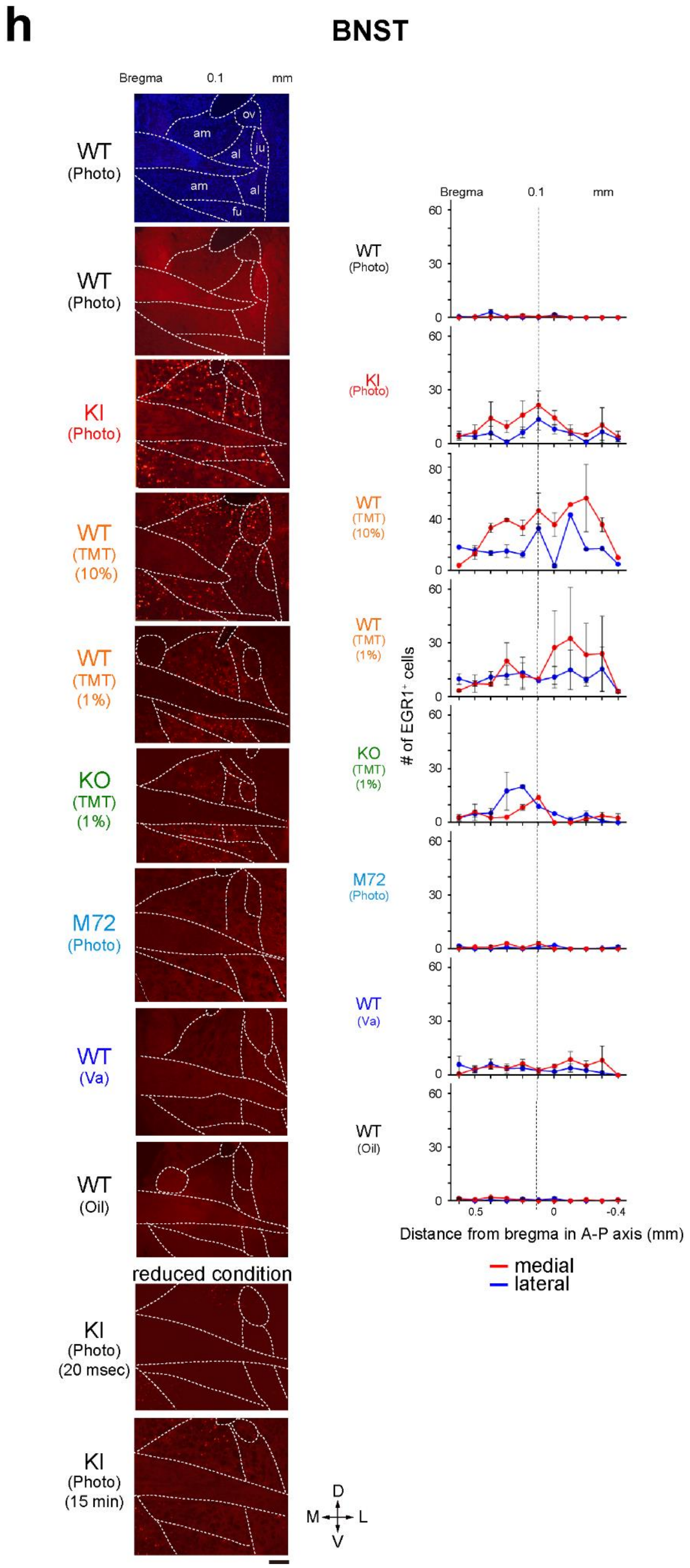

h
BNST
Bregma 0.1 mm
WT (Photo)
am
ov
al
ju
am
al
fu
WT (Photo)
KI (Photo)
WT (TMT) (10%)
WT (TMT) (1%)
KO (TMT) (1%)
M72 (Photo)
WT (Va)
WT (Oil)
reduced condition
KI (Photo) (20 msec)
KI (Photo) (15 min)
D
M
L
V
Bregma 0.1 mm
# of EGR1+ cells
Distance from bregma in A-P axis (mm)
0.5
0
-0.4
medial
lateral

**Supplementary Figure. 4 | Immunohistochemistry for Egr1 expression.** Olfr1019KI, WT, M72-ChR2-YFP mice were photo-illuminated with 2 Hz light of 250 msec pulses. Olfr1019-KO and WT mice were exposed to 1% TMT, 10% TMT, and 10% vanillin (Va). The Olfr1019-KI mice were also treated with reduced condition in photo-illumination (2 Hz light of “20 msec” pulses for 30 min and 2 Hz light of 250 msec pulses for “15 min”. (**a**) A schematic diagram of the mouse OC. (**b**) Egr1 expression in the OB. The Olfr1019-KI glomeruli are labeled with Venus in green. Egr1 signals are shown in red. Scale bars are 250 μm. GL, glomerular layer; EPL, external plexiform layer; MCL, mitral cell layer; and GCL, granule cell layer. Egr1 expression was also analyzed in the following regions: (**c**) AON, anterior olfactory nucleus; (**d**) APC, anterior piriform cortex; (**e**) OT, olfactory tubercle, (**f**) CoA, cortical amygdala; (**g**) MeA, medial amygdala; and (**h**) BNST, bed nucleus of stria terminals. All sections were counterstained with DAPI (**c**-**h**) to identify brain structures microscopically according to the Paxinos and Franklin’s mouse brain atlas. Egr1 signals were quantified for the AON (**c**), APC (**d**), OT (**e**), CoA (**f**), MeA (**g**), and BNST (**h**). (n=5). Dorsal part, medial part, lateral part, and ventral part of the AON (d, m, l v); 1,2,3, layer 1, 2, and 3; cor, cortical zone; Icj, islands of Calleja; anterior, lateral posterior, and medial posterior cortical amygdala (CoA a, CoA pl, CoA pm); cortex amygdala transition zone (CxA); amygdalo-piriform transition area (AmPir); anterior amygdaloid area (AA); basomedial amygdalar nucleus (BMA); basolateral amygdaloid nucleus (BLA); central amygdala (CeA); piriform cortex (PIR); Anterodorsal, anteroventral, posterodorsal, and posteroventral medial amygdala (MeAad, MeAav, MeApd, and MeApv); anterolateral (al); anteromedial (am); fusiform nucleus (fu); juxta capsular nucleus (ju); oval nucleus (ov). Scale bars are 250 μm.

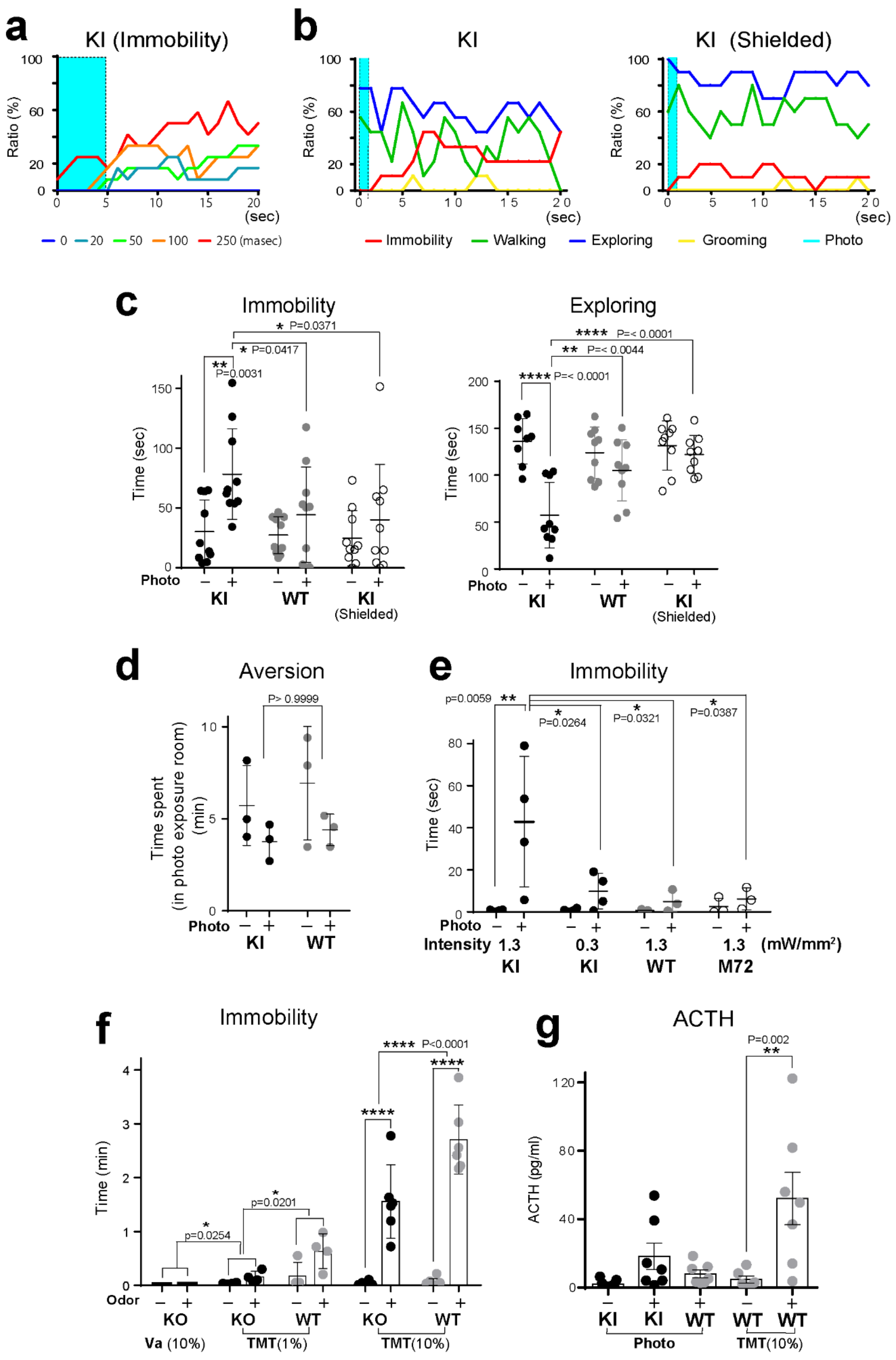

a
KI (Immobility)
Ratio (%)
(sec)
0 20 50 100 250 (masec)
b
KI
KI (Shielded)
Immobility Walking Exploring Grooming Photo
c
Immobility
Exploring
Time (sec)
P=0.0371
P=0.0417
P=0.0031
P=< 0.0001
P=< 0.0044
Photo
KI
WT
KI (Shielded)
d
Aversion
Time spent (in photo exposure room) (min)
P> 0.9999
e
Immobility
p=0.0059
P=0.0264
P=0.0321
P=0.0387
Intensity 1.3 0.3 1.3 1.3 (mW/mm2)
KI KI WT M72
f
Immobility
Time (min)
p=0.0254
p=0.0201
P<0.0001
Odor
KO KO WT KO WT
Va (10%) TMT(1%) TMT(10%)
g
ACTH
ACTH (pg/ml)
P=0.002
KI KI WT WT WT
Photo TMT(10%)

**Supplementary Figure. 5 Behavioral analyses of photo-illuminated KI and TMT exposed KO mice.**

(**a**) Analyzing the immobility behavior in the different durations of photo-illumination. (**b**)Time-course studies of photo-stimulated KI and shielded KI mice. For photo-illumination, 2 Hz light of 250 msec pulses for 1 sec (cyan) was given every 20 sec. Ratios of various behaviors are shown. n=9. (**c**) Immobility and exploratory behaviors. The KI, WT, and shielded KI mice were analyzed. One-sec of photo-stimulation was given to the mice in every 20 sec. Time spent (sec) for each behavior within 3 min after illumination is shown. Asterisks indicate two-way ANVOA followed by Bonferroni correction. **p* <0.05, ** *p* <0.01, *** *p* <0.001, **** *p* <0.0001. Error bars are ±SD. n=9. (**d**) Open-field aversion analyses. Photo-stimulation for 1 sec was given to the mice every 20 sec. KI and WT mice were photo-illuminated in one particular room out of two in a cage. Total time that each animal spent within 10 min in the room where photo-illumination was given (n=3). (**e**) Immobility responses to photo-illumination. Olfr1019-KI WT and M72-YFP-ChR2 (M72) mice were analyzed. Olfr1019-KI with lower intensity (0.3 $mW/mm^2$) were also analyzed. Photo-illumination of 2 Hz for 5 sec with a pulse of 250 msec was given every min. Time spent for immobility during a 3-min period of observation was measured. Asterisks indicate two-way ANVOA followed by Bonferroni correction. **p* <0.05, ** *p* <0.01. Error bars are ±SD. n=4 (KI), 3 (WT), and 3 (M72). (**f**) Immobility responses to 1% and 10% TMT are compared between Olfr1019-KO and WT mice. n=4 (1% TMT) and n=5 (10% TMT). Asterisks indicate two-way ANVOA followed by Bonferroni correction. **p* <0.05, ** *p* <0.01, **** *p* <0.0001. Error bars are ±SD. (**g**) Quantification of plasma ACTH induced by photo-illumination and TMT exposure. Plasma ACTH was measured in WT and KI mice. n=7. Average concentrations of plasma ACTH (pg/ml ±SEM) are shown. *P* values of one-way ANVOA followed by Bonferroni correction are indicated: **p* <0.05, ** *p* <0.01, **** *p* <0.0001.

**a**

| day 1 | day 2 | day 3 | day 4 | day 5 |
|---|---|---|---|---|
| Olfr. (freq.) | Olfr. (freq.) | Olfr. (freq.) | Olfr. (freq.) | Olfr. (freq.) |
| *786* (1) | *1019* (2) | *30* (4) | *521* (4) | *1028* (3) |
| *211* (1) | *433* (2) | *1028* (4) | *786* (2) | *57* (2) |
| | *521* (2) | *876* (3) | *1123* (2) | *521* (2) |
| | *1123* (1) | *786* (2) | *1028* (2) | *211* (1) |
| | *211* (1) | *521* (2) | *57* (1) | *433* (1) |
| | | *1009* (2) | *448* (1) | *448* (2) |

**b**

| No. | Olfr. | No. | Olfr. | No. | Olfr. | No. | Olfr. | No. | Olfr. | No. | Olfr. | No. | Olfr. |
|---|---|---|---|---|---|---|---|---|---|---|---|---|---|
| 1 | *1048* | 39 | *920* | 77 | *1030* | 115 | *365* | 153 | *1002* | 191 | *432* | 229 | *342* |
| 2 | *1052* | 40 | *923* | 78 | *1031* | 116 | *395* | 154 | *1010* | 192 | *433* | 230 | *344* |
| 3 | *1054* | 41 | *957* | 79 | *1032* | 117 | *406* | 155 | *1023* | 193 | *435* | 231 | *345* |
| 4 | *1056* | 42 | *969* | 80 | *1034* | 118 | *410* | 156 | *1028* | 194 | *437* | 232 | *346* |
| 5 | *1104* | 43 | *974* | 81 | *1036* | 119 | *478* | 157 | *1035-ps1* | 195 | *438* | 233 | *347* |
| 6 | *1106* | 44 | *1019* | 82 | *1043* | 120 | *483* | 158 | *1046* | 196 | *441* | 234 | *348* |
| 7 | *1154* | 45 | *1044* | 83 | *1045* | 121 | *484* | 159 | *1062* | 197 | *448* | 235 | *350* |
| 8 | *1155* | 46 | *1045* | 84 | *1086* | 122 | *488* | 160 | *1079* | 198 | *477* | 236 | *352* |
| 9 | *1176* | 47 | *1047* | 85 | *1170* | 123 | *490* | 161 | *1140* | 199 | *816* | 237 | *378* |
| 10 | *1339* | 48 | *1497* | 86 | *1357* | 124 | *605* | 162 | *1157* | 200 | *877* | 238 | *380* |
| 11 | *1377* | 49 | *23* | 87 | *1361* | 125 | *739* | 163 | *1160* | 201 | *895* | 239 | *381* |
| 12 | *1404* | 50 | *52* | 88 | *1500* | 126 | *746* | 164 | *1215* | 202 | *922* | 240 | *382* |
| 13 | *1406* | 51 | *141* | 89 | *430* | 127 | *748* | 165 | *1219* | 203 | *933* | 241 | *385* |
| 14 | *3* | 52 | *145* | 90 | *822* | 128 | *767* | 166 | *1225* | 204 | *970* | 242 | *386* |
| 15 | *30* | 53 | *150* | 91 | *815* | 129 | *768* | 167 | *1317* | 205 | *982* | 243 | *389* |
| 16 | *38* | 54 | *247* | 92 | *73* | 130 | *780* | 168 | *1366* | 206 | *1036* | 244 | *979* |
| 17 | *57* | 55 | *368* | 93 | *811* | 131 | *784* | 169 | *1389* | 207 | *1039/1517* | 245 | *392* |
| 18 | *807* | 56 | *420* | 94 | *1358* | 132 | *792* | 170 | *1410* | 208 | *1133* | 246 | *393* |
| 19 | *133* | 57 | *446* | 95 | *965* | 133 | *798* | 171 | *1449* | 209 | *1156* | 247 | *394* |
| 20 | *134* | 58 | *456* | 96 | *16* | 134 | *805* | 172 | *1496* | 210 | *1167* | 248 | *395* |
| 21 | *139* | 59 | *506* | 97 | *480* | 135 | *809* | 173 | *1451* | 211 | *1168* | 249 | *531* |
| 22 | *143* | 60 | *521* | 98 | *19* | 136 | *812* | 174 | *1510* | 212 | *1328/1519* | 250 | *527* |
| 23 | *146* | 61 | *510* | 99 | *918* | 137 | *813* | 175 | *1512* | 213 | *1329* | 251 | *845* |
| 24 | *147* | 62 | *523* | 100 | *1510* | 138 | *821* | 176 | *814* | 214 | *1362* | 252 | *862* |
| 25 | *160* | 63 | *810* | 101 | *1511* | 139 | *876* | 177 | *20/21* | 215 | *1395* | 253 | *894* |
| 26 | *763* | 64 | *960* | 102 | *151* | 140 | *881* | 178 | *50* | 216 | *1396* | 254 | *898* |
| 27 | *355* | 65 | *937* | 103 | *482* | 141 | *884* | 179 | *62* | 217 | *1403* | 255 | *900* |
| 28 | *411* | 66 | *938* | 104 | *1162* | 142 | *919* | 180 | *74* | 218 | *1408* | 256 | *944* |
| 29 | *424* | 67 | *958* | 105 | *43/403* | 143 | *926* | 181 | *146* | 219 | *1445* | 257 | *980* |
| 30 | *473* | 68 | *959* | 106 | *49* | 144 | *934* | 182 | *218* | 220 | *1504* | 258 | *1022* |
| 31 | *481* | 69 | *973* | 107 | *54* | 145 | *935* | 183 | *239* | 221 | *55* | 259 | *1058* |
| 32 | *508* | 70 | *994* | 108 | *132* | 146 | *961* | 184 | *332* | 222 | *312* | 260 | *1061/1515* |
| 33 | *738* | 71 | *987* | 109 | *221* | 147 | *972* | 185 | *376* | 223 | *1* | 261 | *1065* |
| 34 | *749* | 72 | *988* | 110 | *313* | 148 | *976* | 186 | *414* | 224 | *228* | 262 | *1066* |
| 35 | *826* | 73 | *1009* | 111 | *338* | 149 | *978* | 187 | *419* | 225 | *255* | 263 | *1076* |
| 36 | *875* | 74 | *1020* | 112 | *348* | 150 | *390* | 188 | *421* | 226 | *339* | 264 | *1089* |
| 37 | *878* | 75 | *1026* | 113 | *350* | 151 | *984* | 189 | *426* | 227 | *340* | 265 | *1090* |
| 38 | *904* | 76 | *1029* | 114 | *364-ps1* | 152 | *992* | 190 | *429* | 228 | *341* | 266 | *1352* |

**Supplementary Table 1 | Lists of OR gene clones.**

(**a**) OR clones isolated from DiI-labeling. Isolation frequencies are shown for each *OR* gene clone. ORs that responded to 1mM TMT are marked in red. (**b**) *OR* gene clones analyzed by highthroughput screening. ORs that responded to 1 mM TMT are marked in red (dorsal-zone clones) or blue (ventral-zone clones).
